# *ClSCPL50* Is Associated with Waterlogging-Induced Shoot Elongation in Watermelon as Revealed by BSA-Seq and Transcriptome Profiling

**DOI:** 10.3390/plants15111686

**Published:** 2026-05-29

**Authors:** Xiaoxiao Guan, Ye Wang, Chengchuang Huang, Tingting Xu, Aoyang Lei, Zhongyuan Hu

**Affiliations:** 1 Laboratory of Vegetable Germplasm Innovation and Molecular Breeding, College of Agriculture and Biotechnology, Zhejiang University, Hangzhou 310058, China; 22016161@zju.edu.cn (X.G.); 22316041@zju.edu.cn (Y.W.); 22516069@zju.edu.cn (C.H.); 12516075@zju.edu.cn (T.X.); 22316136@zju.edu.cn (A.L.); 2 Hainan Institute of Zhejiang University, Yazhou Bay Science and Technology City, Sanya 572025, China

**Keywords:** watermelon, waterlogging, shoot elongation, *ClSCPL50*

## Abstract

Waterlogging, like other abiotic stresses, severely inhibits the growth and development of watermelon. However, few genes conferring waterlogging escape response have been identified in this crop to date. In this study, we performed bulked segregant analysis on a F_2_ population exhibiting significant variation in waterlogging-induced shoot elongation. A 6.59 Mb candidate region on chromosome 7 was identified to be associated with waterlogging-induced shoot elongation. Integrated resequencing, fine-mapping and transcriptome analyses indicated that *Cla97C07G134400* (*ClSCPL50*) candidate gene associated with waterlogging-induced shoot elongation. These findings provide novel insights into the genetic basis of the waterlogging escape response in watermelon and identify a potential target gene for improving waterlogging escape response in other dryland crops.

## 1. Introduction

With global warming and climate change intensifying worldwide, waterlogging has emerged as some of the most severe challenges facing agriculture. Waterlogging significantly impacts plant growth by depriving roots of oxygen, result in stunted growth, yellowing of leaves, and even plant death [1]. Enhancing waterlogging tolerance in crops through the development of new, well-adapted varieties represents a promising and sustainable strategy for increasing plant productivity in waterlogged soils.

During evolution, plants have developed various strategies to modify their morphological structures in response to waterlogging stress. Studies have identified two primary strategies employed by crops to adapt to flooding conditions: the Low Oxygen Quiescence Syndrome (LOQS) and the Low Oxygen Escape Syndrome (LOES) [2]. The Low Oxygen Quiescence Syndrome (LOQS) is characterized by a temporary arrest of cellular metabolism and growth, along with suppression of stem elongation to reduce nutrient consumption and prolong plant survival. This strategy is more advantageous under short-term flooding conditions, as it allows carbohydrates to be conserved for essential processes that sustain viability. In the quiescence strategy, the accumulation of ethylene under flooding conditions induces the expression of SUB1A in rice [3]. Under waterlogging stress, SUB1A leads to an increase in brassinosteroid (BR) levels. In turn, BR reduces gibberellin (GA) levels by activating the transcription of the GA metabolic gene GA2ox7, thereby suppressing the GA-mediated promotion of stem elongation [4].

The Low Oxygen Escape Syndrome (LOES) is characterized by rapid elongation of internodes or leaves, enabling plants to reach the water surface and gain access to more oxygen. Studies have shown that the more flood-tolerant *Rumex palustris* exhibits more pronounced petiole elongation and higher petiole aeration under waterlogging stress compared with the less tolerant *Rumex acetosa*. The petioles and roots of *Rumex palustris* contain abundant aerenchyma to facilitate internal gas exchange, which benefits biomass accumulation [5]. In the escape strategy, the ethylene-responsive transcription factor OsEIL1b binds to the promoters of SK1 and SK2 and activates their expression under waterlogging stress in rice, thereby promoting GA-mediated internode elongation [6]. Furthermore, another ethylene-responsive transcription factor, OsEIL1a, enhances GA accumulation and internode elongation by activating the expression of SEMIDWARF1 (SD1s), a gene encoding a GA biosynthetic enzyme [7].

In the wetland plant watercress (*Nasturtium officinale*), flooding triggers a unique dual response strategy: it exhibits both the internode elongation typical of the escape strategy and the suppression of petiole growth characteristic of the quiescence strategy. This opposite molecular regulatory mechanism above and below water provides a valuable model for dissecting the regulatory networks underlying escape and quiescence responses to flooding [8]. Similarly, in rice, a growth promoter ACE1 and a growth repressor DEC1 act antagonistically with GA to regulate internode elongation. This mechanism is conserved in Poaceae and has played an important role during crop domestication [9]. The escape strategy not only accelerates vegetative growth but also induces earlier reproductive growth. In Arabidopsis thaliana, ABA signaling is enhanced under water stress, promoting the translocation of RGLG1/2 from the plasma membrane to the nucleus, where they interact with TOE1/2. This interaction leads to a significant reduction in TOE1/2 protein levels, relieving the repression of flowering genes and thereby inducing flowering [10].

Carboxypeptidases, primarily through their protease or acyltransferase activities, play regulatory roles in various stress responses. Based on their active sites, they are mainly classified into serine carboxypeptidases (SCPs) and metallocarboxypeptidases (MCPs). Under drought stress, the phytohormone abscisic acid (ABA) signaling pathway is activated to regulate stomatal closure. The serine carboxypeptidase-like protein AtSCPL1 in Arabidopsis thaliana functions as a negative regulator of the ABA signaling pathway. The E3 ubiquitin ligase AtAIRP5/GARU promotes the degradation of AtSCPL1, thereby enhancing plant drought tolerance [11]. In Arabidopsis, research on SCPL50 in plant stress responses has primarily focused on its role under low-phosphate stress. In this context, a molecular cascade involving SCPL50 leads to increased expression of the flowering repressor FLC, which significantly delays flowering time, thereby prioritizing plant survival [12].

Watermelon (*Citrullus lanatus* L.) is an important crop that is sensitive to flooding due to its shallow root structure and high oxygen demand [13]. Therefore, it is necessary to study the cellular mechanisms behind watermelon’s resistance to waterlogging. Unfortunately, there are few studies on the molecular mechanisms of watermelon resistance. In this study, we generated segregating populations, including F_2:3_ from crosses between varieties exhibiting significant differences in waterlogging-induced shoot elongation. A bulked segregant analysis (BSA) was employed to identify genes associated with waterlogging-induced shoot elongation. Using competitive allele-specific PCR (KASP) markers, a single major quantitative trait locus (QTL) on chromosome 7 was identified. Furthermore, Transcriptomic differential expression analysis of the candidate genes *ClSCPL50*. Our findings not only provide novel insights into the genetic regulatory mechanisms underlying waterlogging escape in watermelon but also offer a theoretical basis and technical support for molecular design breeding aimed at improving waterlogging escape response in other crops.

## 2. Results

### 2.1. Segregating Population Construction and Genetic Analysis of Waterlogging-Induced Plant Height

**Figure 1:**
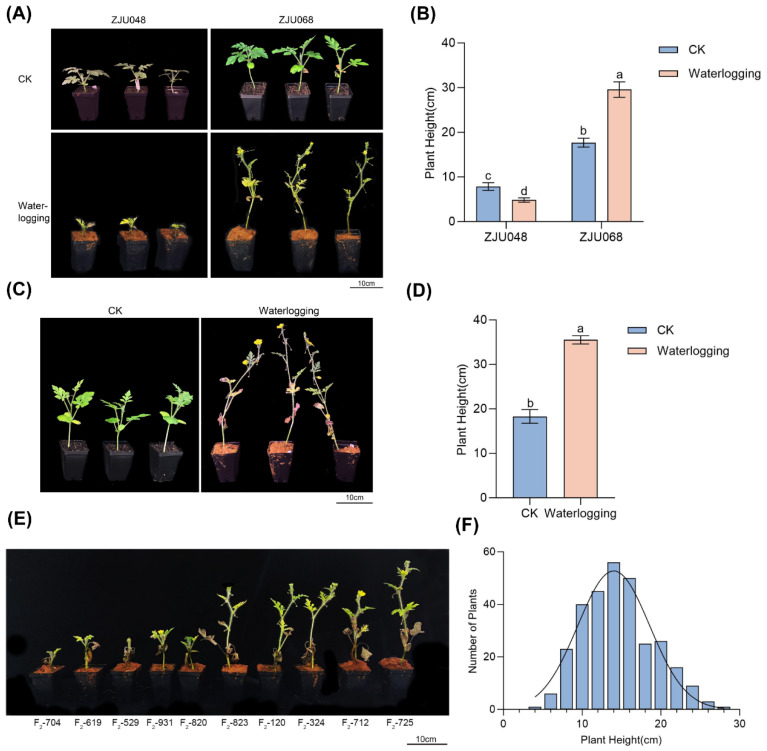
Phenotypic analysis and inheritance of waterlogging escape response in ZJU048 and ZJU068. (**A**) Phenotypes of ZJU048 and ZJU068 after 14 days of control and waterlogging treatments; (**B**) Plant height of ZJU048 and ZJU068 after 14 days of control and waterlogging treatments, *p* < 0.05, Data are mean ± SD; lowercase letters indicate a significant difference determined by one-way ANOVA test; (**C**) Phenotypes of the F1 generation after 14 days of control and waterlogging treatments; (**D**) Plant height of the F1 generation after 14 days of control and waterlogging treatments, *p* < 0.05, Data are mean ± SD; lowercase letters indicate a significant difference determined by Student’s *t*-test; (**E**) Extreme phenotype individuals of the F_2_ generation after 14 days of waterlogging treatment; (**F**) Frequency distribution of plant height in the F_2_ generation of 295 individuals.

Using ZJU048 and ZJU068 as experimental materials, plants at the two-leaf-one-heart stage were subjected to either normal watering (control) or continuous waterlogging treatment for 14 days. Under waterlogging, ZJU048 exhibited restricted growth and adopted a quiescence strategy, whereas ZJU068 displayed accelerated growth and adopted an escape strategy (Figure 1A). Shoot heights of ZJU048 and ZJU068 were measured under both control and waterlogged conditions. Under normal watering, ZJU068 (17.69 cm) was 1.25-fold taller than ZJU048 (7.84 cm). After 14 days of waterlogging stress, the growth of ZJU048 (4.83 cm) was significantly inhibited. In contrast, ZJU068 (29.58 cm) showed rapid stem elongation following waterlogging (Figure 1B). Under waterlogging, ZJU048 exhibited a 38.4% reduction in plant height (7.84 cm → 4.83 cm), whereas ZJU068 exhibited a 67.2% increase (17.69 cm → 29.58 cm). Collectively, ZJU068 exhibited faster and more vigorous shoot growth than ZJU048 under waterlogged conditions, suggesting that the two genotypes display opposite shoot height response patterns to waterlogging, likely due to genetic differences. The F_1_ progeny derived from crossing ZJU048 (male parent) with ZJU068 (female parent) were also subjected to waterlogging treatment. The F_1_ plants exhibited a phenotype similar to the female parent (ZJU068), with accelerated growth after waterlogging (Figure 1C). This observation is consistent with a dominant mode of inheritance for the waterlogging-induced stem elongation trait; however, because reciprocal crosses were not performed, maternal or cytoplasmic effects cannot be formally excluded at this stage. Statistical analysis of plant height in the F_1_ population revealed that under waterlogging, F_1_ plants reached 35.52 cm, approximately twice the height of control plants (18.29 cm) (Figure 1D), demonstrating a pronounced “escape strategy”. Furthermore, in an F_2_ population comprising 295 individuals, plant heights after 14 days of waterlogging treatment ranged from 4.16 cm to 27.07 cm (Figure 1E) and followed a normal distribution (Figure 1F). This distribution indicates that the trait is a quantitative trait controlled by major genes.

### 2.2. Mapping of the QTL Associated with the Waterlogging-Induced Shoot Elongation

High-throughput sequencing was performed on genomic DNA samples from the two parental lines and two extreme phenotype bulks (High-pool for tall plant height and Low-pool for dwarf plant height), generating a total of 42.25 Gb of raw data. After filtering to remove low-quality reads and adapter contamination, 37.23 Gb of clean data were obtained, indicating a high data utilization efficiency. Quality assessment results showed that the Q20 values for all samples exceeded 95.44%, and the Q30 values were no less than 88.96%, demonstrating satisfactory sequencing accuracy and meeting the requirements for downstream analyses. The high Q20 and Q30 values reflect an extremely low sequencing error rate, providing a reliable data foundation for subsequent detection of rare variants such as SNPs and InDels. The GC content of each sample ranged from 35.49% to 37.59%, which is consistent with the genomic characteristics of watermelon and indicates no significant base preference. The clean reads were aligned to the reference genome of watermelon (97103) version 2. The alignment results showed that the mapping rate for each sample was no less than 91.56%, the average sequencing depth ranged from 10.12× to 38.3×, and the percentage of regions covered at least 5× reached or exceeded 80.58% (Table S1). The high mapping rates indicate good sequence homology between the tested materials and the reference genome, while the moderate sequencing depth and high coverage ensure the sensitivity and reliability of variant site detection. Collectively, these results demonstrate that the BSA-seq data obtained in this study are of high quality and suitable for subsequent SNP/InDel detection and association analysis of candidate intervals.

**Figure 2:**
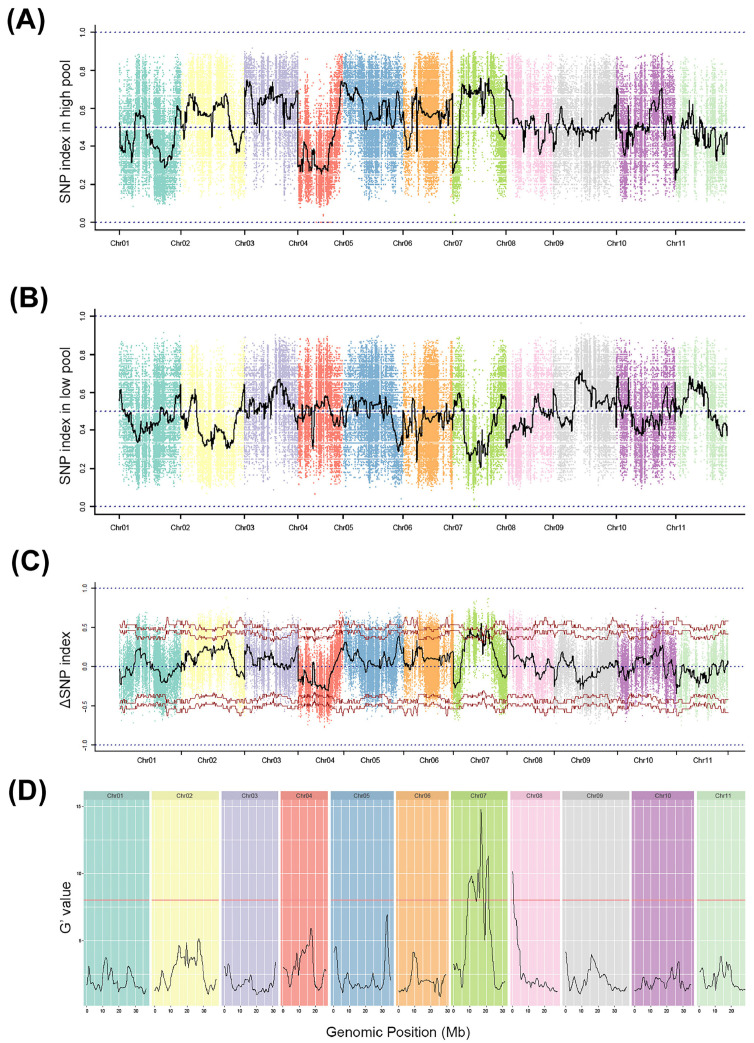
BSA mapping of the plant height trait in watermelon after waterlogging based on BSA-seq and the G’ value. (**A**) Graphs of the SNP-index of the High-pool. The *x* axis represents the 11 chromosomes of watermelon and the *y* axis represents the SNP-index; (**B**) Graphs of the SNP-index of the Low-pool; (**C**) Values of ΔSNP-index for association analysis. The x-axis and y-axis represent the 11 watermelon chromosomes and the SNP index, respectively. The red line indicates the association threshold; (**D**) Identification of QTLs for plant height in watermelon after waterlogging treatment across the whole genome using QTLseqr.

The sequencing reads were aligned to the reference genome using the BWA alignment tool, with ZJU048 and ZJU068 serving as parental references. After stringent filtering and removal of repetitive sequences, the distribution frequency of single nucleotide polymorphisms (SNPs) across the 11 chromosomes was obtained for the progeny, and the SNP-index value was calculated for each SNP site. The SNP-index and InDel-index of the two progeny bulks were combined to obtain the integrated index (All-index) for the High-pool and Low-pool, and the distribution of this index across the 11 watermelon chromosomes was plotted (Figure 2A,B). Furthermore, Δ(All-index) was calculated as the difference in All-index between the two bulks, and its chromosomal distribution was similarly plotted (Figure 2C). At a 99% confidence level, one candidate interval associated with the regulation of watermelon plant height under waterlogging stress was detected. This interval is located on chromosome 7, spanning from Cla97Chr07: 10,475,818 to 17,062,197 bp, with a total length of 6.59 Mb and containing 56 genes.

Using the QTLseqr software (v0.7.5.2), a G’ value distribution map was generated. At the 99% confidence level, two genomic regions exhibited G’ values significantly exceeding the threshold, located on chromosome 7 (Cla97Chr07: 9,524,986–21,967,246 bp) and chromosome 8 (Cla97Chr08: 404–89,885 bp), respectively. The region identified by BSA was mutually validated by QTL-seq analysis (Figure 2D). Based on these results, the intersection of the intervals identified by the two algorithms was taken, and the region on chromosome 7 (Cla97Chr07: 10,475,818–17,062,197 bp) was preliminarily designated as the candidate genomic region controlling watermelon plant height under waterlogging stress. Subsequent fine mapping and functional characterization will focus on the genes within this interval.

### 2.3. Fine Mapping of the Candidate Gene

Based on the preliminary mapping of genomic regions associated with the regulation of watermelon plant height under waterlogging stress, fine mapping was further conducted within the candidate interval. A total of 10 single nucleotide polymorphism (SNP) sites located within this interval were designed and selected, designated as M1 through M10, and corresponding Kompetitive Allele-Specific PCR (KASP) markers were subsequently developed. Genotyping of the segregating population was performed using these markers to narrow down the candidate region.

**Figure 3:**
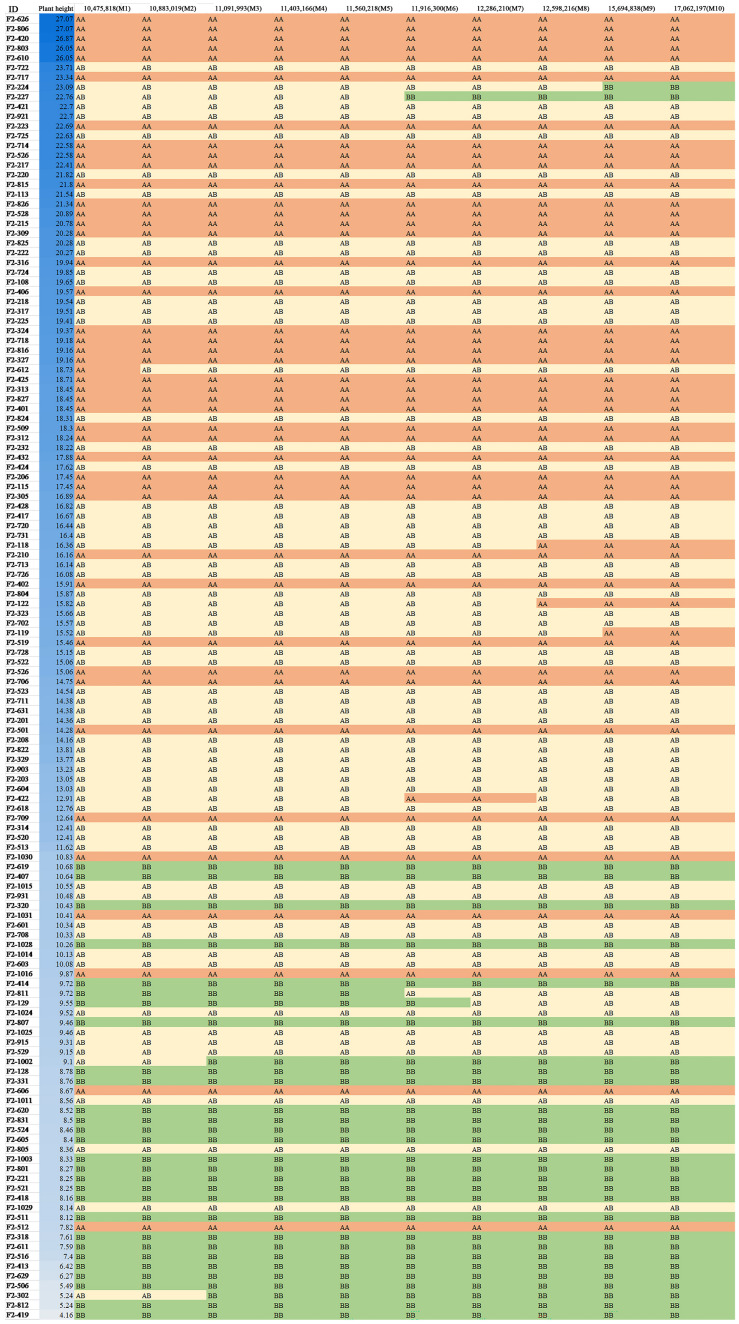
Haplotype origin analysis of candidate intervals. Orange indicates AA (ZJU068 homozygous genotype) and BB (ZJU048 homozygous genotype); yellow indicates the heterozygous region. Numbers in the first row (e.g., 10,475,818, 10,883,190, etc.) indicate the physical genomic positions (bp) of each marker on chromosome 7. Marker labels M1–M10 are shown alongside the corresponding genomic coordinates.

The genotypes of the selected SNP markers were converted into a schematic representation of chromosome haplotype origins. When the 134 F_2_ individuals were arranged in descending order according to plant height after waterlogging treatment, the results showed that most homozygous individuals carrying the ZJU068 genotype (AA) were clustered at the top, those carrying the ZJU048 genotype (BB) were predominantly clustered at the bottom, while heterozygous individuals (AB) were clustered in the middle region (Figure 3). These data further demonstrate a strong association between the genotype of the candidate region and the waterlogging-induced plant height phenotype in watermelon.

**Figure 4:**
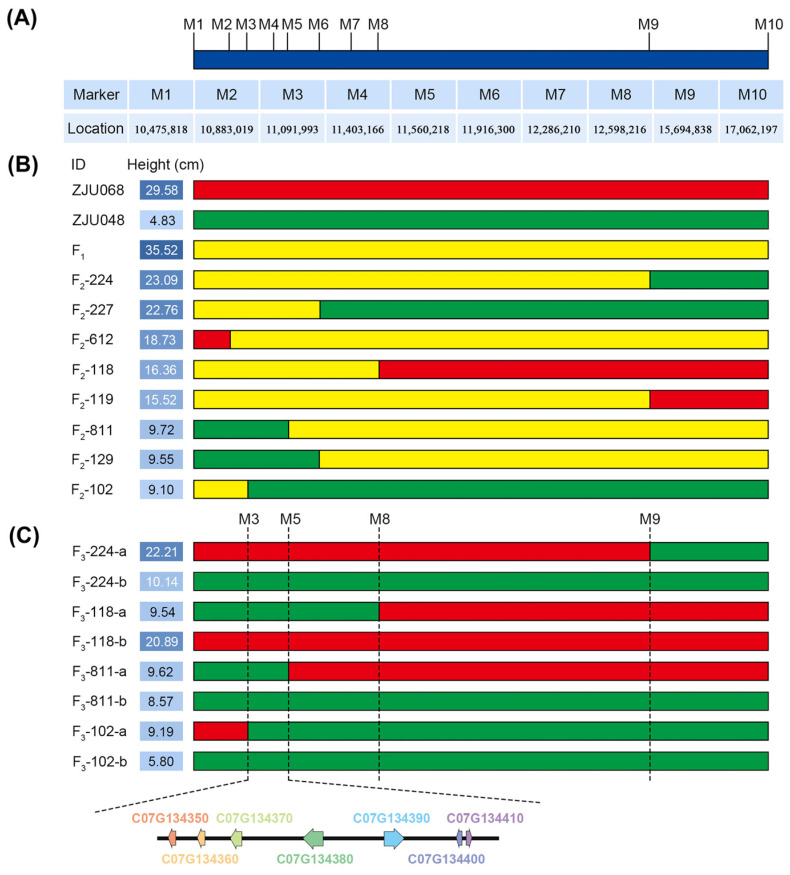
Fine mapping of candidate genes regulating watermelon plant height after waterlogging. (**A**) There are recombinant individuals with chromosome segment substitutions in the preliminary candidate interval. Red indicates segments from homozygous ZJU068, green indicates segments from homozygous ZJU048, and yellow indicates heterozygous regions; (**B**) The self-pollinated progenies of the recombinant individuals F_2_-224, F_2_-118, F_2_-811, and F_2_-102 were divided into two groups (a and b) based on their chromosome origin, and the difference in plant height after waterlogging between the two groups was analyzed. Plant height was measured as the average height from three F3 progeny individuals; (**C**) Candidate genes in the target region.

Among the 295 F_2_ segregating population individuals, 28 plants exhibited chromosomal recombination events within the candidate interval. Analysis of recombination patterns in relation to genotypes revealed that the candidate interval could be narrowed to M2–M6 based on recombination events in F_2_ individuals with greater waterlogging-induced plant height. Further refinement to M3–M5 was achieved by incorporating recombination events from F_2_ individuals with reduced plant height after waterlogging (Figure 4A). Using the F_3_ progeny derived from recombinant individuals, the self-pollinated offspring of F_2_-118 were divided into two groups (F_2_-118-a and F_2_-118-b) based on their genotypes. These two groups showed significantly different plant heights after waterlogging (Figure 4B), indicating that the heterozygous region of the recombinant F_2_-118 individual contains candidate genes regulating waterlogging-induced plant height in watermelon, i.e., the region between markers M1 and M8. For the self-pollinated offspring of F_2_-811 (F_2_-811-a and F_2_-811-b), no significant difference in plant height was observed after waterlogging, suggesting that the homozygous region of F_2_-811, namely the interval between M1 and M5, harbors candidate genes involved in the regulation of waterlogging-induced plant height. Meanwhile, the self-pollinated progeny of recombinant individual F_2_-102 (F_2_-102-a and F_2_-102-b) also showed no significant difference in plant height after waterlogging, indicating the absence of relevant candidate genes in its heterozygous region and allowing the exclusion of the M1–M3 interval. Comprehensive analysis of the F_3_ segregating populations consistently supported the interval M3–M5 (11,091,993–11,560,218 bp) as the candidate region controlling waterlogging-induced plant height in watermelon.

KASP markers developed at regular intervals were used for genotypic identification and phenotypic evaluation of F_2_ individuals and their corresponding F_3_ family populations. Joint analysis further narrowed the candidate interval to the region between markers M3 and M5, spanning 468.2 kb. According to the Chinese Watermelon Genome Database, this interval contains a total of seven candidate genes (Figure 4C).

### 2.4. Expression Profiling of Candidate Genes Within the Fine-Mapped Interval

**Figure 5:**
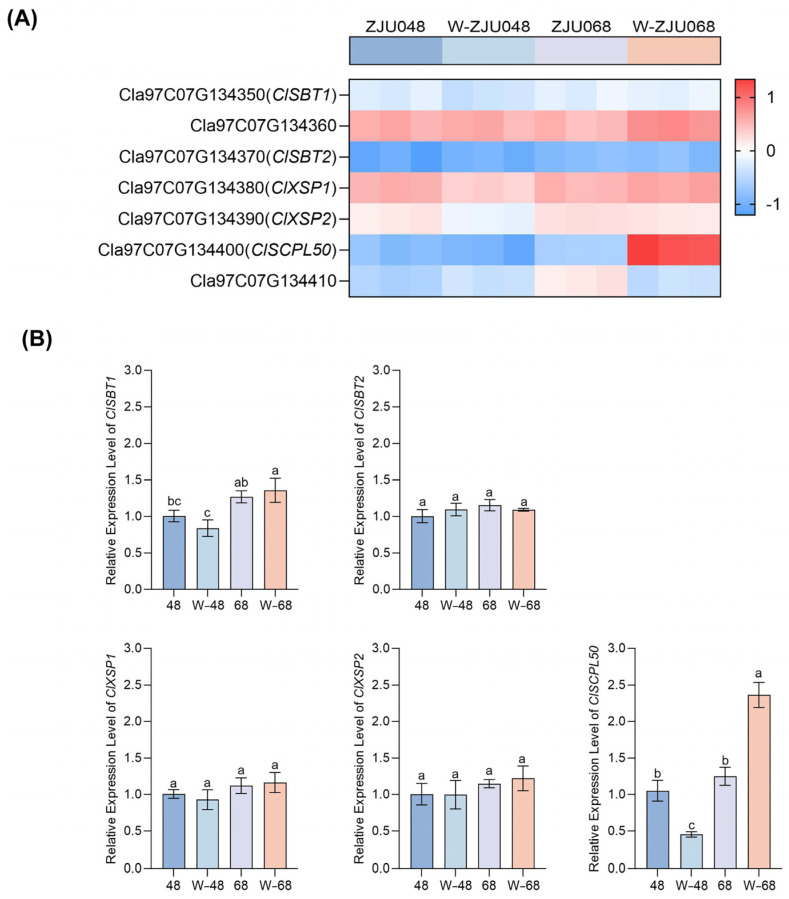
Candidate interval gene expression profiling under waterlogging treatment. ‘W’ stands for waterlogging treatment; W-ZJU048 and W-ZJU068 represent waterlogging treatment conditions. (**A**) Heatmap of differential transcriptome expression of genes within the candidate interval; (**B**) Analysis of gene expression levels within the candidate interval, lowercase letters indicate a significant difference determined by one-way ANOVA test (*p* < 0.05).

Visualization of the expression levels of the seven genes within the candidate interval based on RNA-seq data, presented as a heatmap, revealed distinct response patterns between parental lines and treatments (Figure 5A). No significant changes in expression were observed for *Cla97C07G134350*, *Cla97C07G134360*, *Cla97C07G134370*, and *Cla97C07G134380* between the two parental lines or between control and waterlogging conditions. In the waterlogging-sensitive accession ZJU048, the transcript level of *Cla97C07G134390* decreased after waterlogging stress compared with the control. *Cla97C07G134410* showed upregulation only in the waterlogging-tolerant accession ZJU068 under normal growth conditions, with no similar trend observed after waterlogging. In contrast, *Cla97C07G134400* exhibited an expression pattern closely associated with waterlogging escape response: waterlogging treatment suppressed its expression in ZJU048 but induced its expression in ZJU068. This finding suggests that waterlogging stress may act as an upstream signal, differentially regulating the expression of this gene in opposite directions depending on the genetic background.

Quantitative real-time PCR (qRT-PCR) validation of the annotated genes within the candidate interval (Figure 5B) further clarified their expression patterns. Among them, *Cla97C07G134350* (*ClSBT1*) showed differential expression between ZJU048 and ZJU068, but waterlogging treatment did not markedly affect its transcript levels. No significant changes in expression were detected for *Cla97C07G134370* (*ClSBT2*), *Cla97C07G134380* (*ClXSP1*), and *Cla97C07G134390* (*ClXSP2*) across all experimental groups. In comparison, the expression trend of *Cla97C07G134400* (*ClSCPL50*) was highly consistent with the RNA-seq data. The enzyme encoded by this gene is known to be involved in cell wall loosening and hormone metabolism pathways. Based on these expression characteristics, *ClSCPL50* is proposed as a key candidate gene regulating stem elongation in watermelon under waterlogging stress, with its expression level positively correlated with the intensity of the response strategy adopted by the plant.

### 2.5. Sequence Variation Analysis of Candidate Genes ClSCPL50

**Figure 6:**
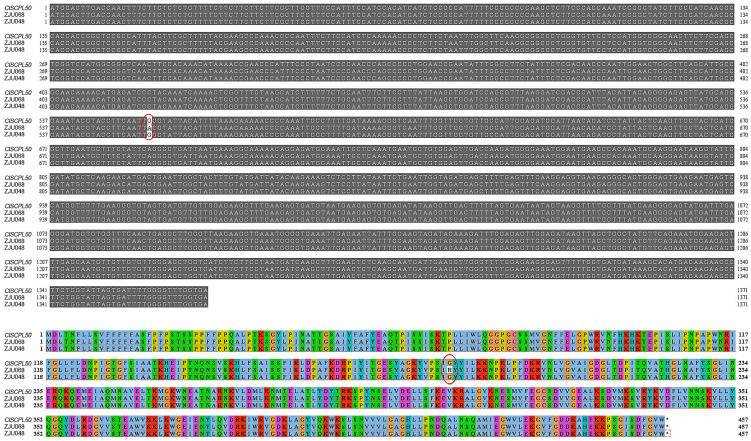
Alignment of CDS and amino acid sequences of *ClSCPL50* in ZJU048 and ZJU068. The red circles indicate the positions of the variant bases and amino acids. The asterisk * represents the stop codon.

Analysis of the BSA sequencing data revealed a single nucleotide polymorphism (C → T) in the candidate gene *Cla97C07G134400* (*ClSCPL50*). This gene consists of a single exon in both parental lines. Cloning and sequencing of the coding sequences (CDS) from the two parents identified a single base mutation (G → A) at position 556 of the CDS of ZJU068. This nucleotide change results in an amino acid substitution at position 186 of the encoded protein, converting glycine (G) to arginine (R) (Figure 6). Therefore, it is postulated that this amino acid substitution, caused by the single base mutation, may alter the function of the SCPL50 protein, leading to phenotypic differences in watermelon plant height after waterlogging. To evaluate the potential functional significance of this substitution, we performed domain analysis and in silico prediction. (1) Protein domain analysis: We searched the Pfam database to characterize the domain architecture of *ClSCPL50*. The protein contains a serine carboxypeptidase (SCP) domain (Pfam: PF00450), and residue 186 is located within this conserved catalytic domain (Figure S1). (2) In silico prediction: We used SIFT (Sorting Intolerant From Tolerant) and PolyPhen-2 (Polymorphism Phenotyping) to predict the functional impact of the Gly186Arg substitution. The SIFT score was 0.04 (deleterious, threshold < 0.05) and the PolyPhen-2 score was 0.999 (deleterious, threshold > 0.8), both predicting that this substitution is likely to affect protein function. Therefore, it is postulated that this amino acid substitution, caused by the single base mutation, may alter the function of the SCPL50 protein, leading to phenotypic differences in watermelon plant height after waterlogging.

### 2.6. Association Analysis of ClSCPL50 Genotyping with Waterlogging-Induced Shoot Elongation

**Figure 7:**
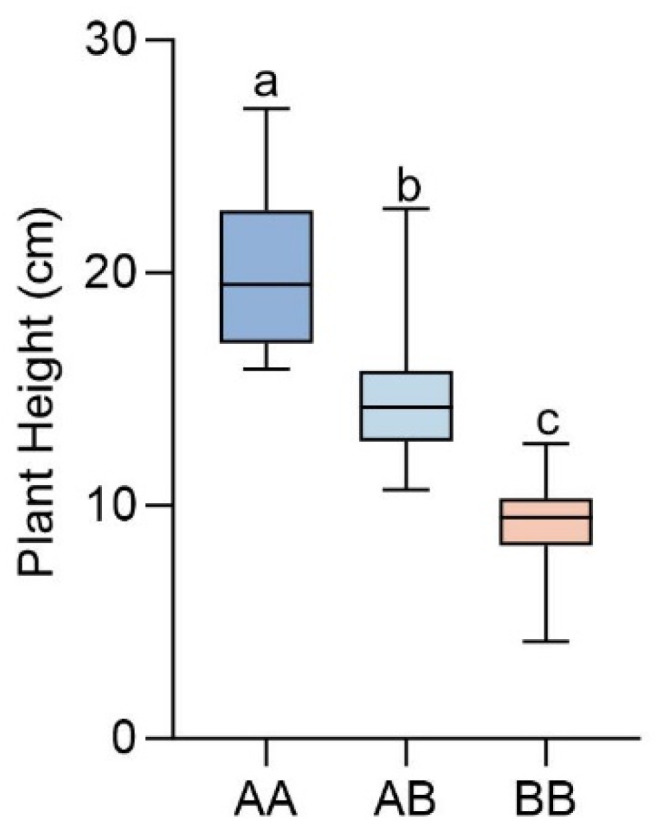
Relationship between the distribution of *ClSCPL50* alleles and watermelon plant height after waterlogging in the F_2_ generation. AA (*n* = 63), AB (*n* = 156), and BB (*n* = 76). Data are mean ± SD; lowercase letters indicate a significant difference determined by one-way ANOVA test (*p* < 0.05).

To further dissect the genetic effect of the candidate gene *ClSCPL50*, a KASP marker M5 (Chr07-11560218) was developed in the vicinity of this gene for genotyping the population materials. The three genotypes AA, AB, and BB correspond to the ZJU068 homozygous, heterozygous, and ZJU048 homozygous types, respectively. In the F_2_ population comprising 295 individuals, individuals carrying the AA genotype exhibited significantly greater plant height after waterlogging treatment than those with the AB genotype, and AB individuals in turn showed significantly greater plant height than BB individuals (Figure 7). These results confirm that *ClSCPL50* is involved in the regulation of watermelon plant height under waterlogging conditions, supporting its role as a major gene affecting waterlogging-induced plant height in watermelon.

## 3. Discussion

Watermelon is one of the most economically important crops worldwide, with a cultivation area covering millions of hectares and an annual output exceeding 100 million tons (https://www.fao.org/). However, as a xerophytic crop, watermelon is highly sensitive to waterlogging. In recent years, due to the frequent occurrence of extreme rainfall and typhoons, many watermelon-growing regions have been repeatedly affected by waterlogging stress, resulting in inhibited plant growth and a sharp decline in both yield and fruit quality. Therefore, enhancing waterlogging escape response has become a critical challenge that urgently needs to be addressed in current watermelon breeding and production.

In this study, we systematically characterized the phenotypic responses of two watermelon accessions, ZJU048 and ZJU068, to waterlogging stress and identified a major genetic locus controlling waterlogging-induced shoot elongation. Our results demonstrate that ZJU048 adopts a quiescence strategy characterized by growth restriction, whereas ZJU068 employs an escape strategy manifested by rapid stem elongation. These opposite adaptive strategies within the same species provide a valuable genetic resource for dissecting the molecular mechanisms underlying waterlogging escape response in watermelon. Similar dichotomous responses have been documented in other species, such as rice (*Oryza sativa*) and Rumex species, highlighting the evolutionary conservation of these two contrasting strategies [14,15].

The genetic analysis revealed that waterlogging-induced stem elongation is a dominant trait, as evidenced by the F_1_ progeny exhibiting a phenotype similar to that of the female parent ZJU068. We note, however, that this conclusion regarding dominance is tentative due to the absence of reciprocal crosses; maternal or cytoplasmic effects cannot be formally excluded at this stage. Future studies will include reciprocal crosses to distinguish between true dominance and maternal or cytoplasmic contributions to the inheritance pattern. The normal distribution of plant heights in the F_2_ population further indicates that this trait is a quantitative trait controlled by major genes, which is consistent with previous findings in other crops regarding flooding tolerance [2,9]. For instance, in rice, the SUB1A locus confers a quiescence strategy and behaves as a dominant or semi-dominant trait depending on the genetic background [3,16].

Through BSA-seq and QTL-seq analyses, a 6.59 Mb candidate region on chromosome 7 was initially identified. Fine mapping using newly developed KASP markers narrowed this interval to 468.2 kb between markers M3 and M5, containing seven annotated genes. This stepwise reduction of the candidate interval demonstrates the effectiveness of integrating BSA with high-resolution genetic mapping in populations with recombination events. Similar approaches have been successfully applied to identify flooding-related genes in cucumber and maize [17,18].

Among the seven candidate genes, only *ClSCPL50* (Cla97C07G134400) exhibited expression patterns that correlated strongly with the phenotypic response. RNA-seq and qRT-PCR analyses consistently showed that waterlogging suppressed *ClSCPL50* expression in the quiescent accession ZJU048 but induced its expression in the escape accession ZJU068. This opposite regulation suggests that *ClSCPL50* may act as a molecular switch directing the plant toward either growth arrest or accelerated elongation under waterlogged conditions. The encoded SCPL50 protein belongs to the serine carboxypeptidase-like (SCPL) family, members of which have been implicated in cell wall modification, phytohormone metabolism, and stress signaling [12,19]. In Arabidopsis, *SCPL50* has been shown to participate in low-phosphate stress responses by modulating flowering time through the FLC pathway [12], suggesting functional diversification of SCPL proteins across different stress conditions. Therefore, *ClSCPL50* may modulate waterlogging-induced shoot elongation by altering cell wall extensibility or regulating gibberellin (GA)/brassinosteroid (BR) homeostasis, similar to the roles of *SUB1A*, *ACE1*, and *DEC1* in rice [3,4,9]. Notably, in the rice escape strategy, ethylene-responsive transcription factors OsEIL1a and OsEIL1b activate GA biosynthesis genes such as SD1s and SK1/SK2, promoting internode elongation [6,7]. Whether *ClSCPL50* interacts with ethylene or GA signaling pathways in watermelon warrants further investigation.

A critical nonsynonymous SNP (G → A) was identified in the coding sequence of *ClSCPL50*, resulting in a glycine-to-arginine substitution at position 186 of the protein. This amino acid change is predicted to affect protein function, as supported by domain analysis and in silico predictions. Specifically, residue 186 is located within the conserved serine carboxypeptidase (SCP) catalytic domain (Pfam: PF00450) (Figure S1), and computational tools SIFT (score = 0.04, deleterious threshold < 0.05) and PolyPhen-2 (score = 0.999, deleterious threshold > 0.8) both indicate that the Gly186Arg substitution is likely deleterious. This may explain the phenotypic divergence between ZJU048 and ZJU068. Nonsynonymous mutations in stress-responsive genes have been shown to alter protein stability, enzymatic activity, or substrate specificity, thereby affecting adaptive phenotypes [20,21]. The association analysis using KASP marker M5 further confirmed that the AA genotype (ZJU068 type) is significantly associated with greater plant height after waterlogging, while the BB genotype (ZJU048 type) correlates with growth suppression. These results provide strong genetic evidence supporting *ClSCPL50* as the causal gene underlying waterlogging-induced shoot elongation. We note that the conclusion regarding the dominant inheritance of waterlogging-induced stem elongation is tentative because reciprocal crosses were not performed. Future studies will include reciprocal crosses to formally test for maternal or cytoplasmic contributions to the inheritance pattern. We acknowledge that direct evidence for the functional impact of the Gly186Arg substitution would require biochemical complementation assays and heterologous expression, and that confirmation of the causal role of *ClSCPL50* will ultimately depend on genetic transformation or CRISPR-based validation. These approaches have been explicitly prioritized for future investigation. Interestingly, sequence alignment revealed no polymorphism between the two parental lines in the proximal promoter region of *ClSCPL50* (Figure S2). Therefore, the differential expression of *ClSCPL50* observed under control conditions (Figure 5A) cannot be explained by cis-regulatory variation in the proximal promoter. Instead, other mechanisms may be involved, such as differences in distal regulatory elements, intronic regions, untranslated regions (UTRs), epigenetic modifications (e.g., DNA methylation or histone marks), or post-transcriptional regulation.

It is noteworthy that the other six genes within the fine-mapped interval did not show waterlogging-responsive expression patterns or consistent differences between the two parents. Although we cannot completely exclude their involvement in minor regulatory roles, the cumulative evidence from genetic mapping, expression profiling, sequence variation, and association analysis points to *ClSCPL50* as the most plausible candidate. A similar scenario has been reported in other crops, where a single major gene within a fine-mapped interval determines the differential stress response, while neighboring genes exhibit little or no functional relevance [8,10]. Nevertheless, we recognize that plant height measured after 14 days of waterlogging serves as a proxy trait for the escape response. Future investigations should therefore include additional fitness parameters (e.g., survival rate, post-stress recovery, and final yield) to comprehensively evaluate waterlogging tolerance. Moreover, we explicitly acknowledge that functional validation via genetic transformation and CRISPR-based approaches is required to confirm the causal role of *ClSCPL50*. Accordingly, future work should focus on such functional validation, exploration of downstream target genes of *ClSCPL50*, and elucidation of its interactions with phytohormone signaling pathways under waterlogging stress. Furthermore, comparative analyses between watermelon and other flood-tolerant species, such as *Rumex palustris* and deepwater rice, may reveal conserved and divergent regulatory mechanisms of SCPL proteins in adaptation to low-oxygen conditions.

## 4. Materials and Methods

### 4.1. Plant Materials

The F_1_ progeny was generated by crossing ZJU048 (male parent) with ZJU068 (female parent). The F_1_ plants were strictly self-pollinated to produce the F_2_ population. Recombinant individuals were selected from the F_2_ population, and subsequently self-pollinated to establish the F_3_ lines. All seedlings were grown individually in black plastic pots filled with a mixture of peat, vermiculite, and perlite (3:1:1, *v*/*v*) in a greenhouse maintained at 28 °C during the day and 20 °C at night.

### 4.2. Waterlogging Treatment

For waterlogging treatment, seedlings at the two-true-leaf stage were placed in large blue boxes filled with water (pH 7.03 at 25 °C, electrical conductivity 0.34 dS·m^−1^, dissolved oxygen concentration 8.17 mg·L^−1^). The water level was adjusted to reach the base of the first true leaf. During the treatment period, water was replenished daily to maintain a constant water level, compensating for evaporation and any water loss. After 14 days of waterlogging, plant height (measured from the soil surface to the top of the plant) was recorded for each plant.

### 4.3. DNA Extraction, Quality Detection and Library Construction

Total genomic DNA was extracted from fresh leaves of young plants using the CTAB method (VWI, China) as described in Lyu et al. [22]. DNA degradation and contamination were monitored on 1.5% agarose gels. DNA purity was checked using the NanoPhotometer^®^ spectrophotometer (IMPLEN, Westlake Village, CA, USA) DNA concentration was measured using Qubit^®^ DNA Assay Kit in Qubit^®^2.0 Flurometer (LifeTechnologies, Carlsbad, CA, USA). Two bulked DNA pools were constructed: a long-pool from 20 individuals with the highest seedlings and a short-pool from 20 individuals with the shortest seedlings, selected from the F_2_ population. Genomic DNA from individual seedlings within each phenotypic group (High-pool and Low-pool) was mixed in equal proportions. The DNA samples were then randomly sheared to an average fragment size of 350 bp using a Covaris crusher. Sequencing libraries were prepared with the TruSeq Library Construction Kit (Illumina, San Diego, CA, USA) following the manufacturer’s instructions. The resulting libraries were sequenced on an Illumina HiSeq 4000 platform, generating 150 bp paired-end reads with an insert size of approximately 350 bp. Raw sequencing data quality was assessed using FASTQC (V0.11.9) [23] and subsequent quality control (QC) procedures were implemented using standard pipelines as described by Liao et al. [24].

### 4.4. Variant Calling, Annotation, and BSA

Clean reads from each sample were aligned to the 97,103 reference genome (version 2.0, available at Cucurbit Genomics) using Burrows-Wheeler Aligner (BWA) [25]. The resulting sequence alignment/map (SAM) files were converted to binary alignment/map (BAM) format using SAMtools (V1.10) [26]. Variant calling across all samples was performed with the UnifiedGenotyper function in the Genome Analysis Toolkit (GATK) (V4.6.2.0) [27]. Single nucleotide polymorphisms (SNPs) were identified, and insertions/deletions (InDels) were filtered using the VariantFiltration parameter within GATK. The detected SNPs and InDels were annotated based on the reference genome’s GFF3 file using ANNOVAR (V2020-06-08) software [28]. All homozygous SNPs/InDels that were polymorphic between the two parents were extracted from the variant call format (VCF) file. The read depth information for these homozygous SNPs/InDels in the progeny bulks was retrieved, and the SNP/InDel index was calculated as described by Takagi et al. [29]. Briefly, using the genotype of parent ZJU048 as the reference, we quantified the number of reads supporting this reference allele in the offspring pools. The SNP/InDel index for each site was then calculated as the ratio of reads with the alternative allele to the total number of reads. Sites with an SNP/InDel index of less than 0.3 in both the long and short pools were filtered out. The G’ statistic, a measure of association strength, was subsequently calculated using the QTLseqr package in R, following the methodology outlined by Mansfeld and Grumet [30].

### 4.5. Fine Mapping with KASP Markers

To narrow down the target region identified by BSA-seq, Kompetitive Allele-Specific PCR (KASP) markers were developed based on SNPs within the candidate interval. Corresponding primer sets (Fam-labeled, Hex-labeled, and a common reverse primer) were designed for genotyping the F_2_ and F_2:3_ populations. The KASP assay was performed as previously described by Liao et al. [24]. PCR amplifications were conducted in an Eppendorf Gradient thermal cycler using the following program: Stage 1 (Hot Start): 30 °C for 1 min; 94 °C for 15 min. Stage 2 (Touchdown PCR): 10 cycles of 94 °C for 20 s and 61 °C for 60 s. The annealing temperature was decreased by 0.6 °C per cycle. Stage 3 (Standard PCR): 28 cycles of 94 °C for 20 s and 55 °C for 60 s. Stage 4 (Post-read): 30 °C for 1 min. Following amplification, endpoint fluorescence was detected and analyzed using an ABI PRISM 7900HT system (Applied Biosystems, Foster City, CA, USA) to assign genotypes. All primers used for KASP genotyping are listed in Table S2.

### 4.6. RNA Extraction, Reverse Transcription, and qRT-PCR

Total RNA was extracted using a commercial RNA Extraction Kit (Easy-do, Shenzhen, China). Subsequently, 1 µg of the extracted total RNA was reverse-transcribed into first-strand cDNA using the ReverTra Ace qPCR RT Master Mix (Toyobo, Osaka, Japan). Quantitative real-time PCR (qRT-PCR) was performed with TOROGreen qPCR Master Mix (Toyobo) on an ABI StepOne Plus Real-Time PCR System (Applied Biosystems, Foster City, CA, USA). The expression level of the GAPDH gene (Cla97C02G044620) served as an internal control for normalizing the relative expression of candidate genes, which was calculated using the comparative 2^–ΔΔCt^ method [31]. All primers used for qRT-PCR are listed in Table S3.

### 4.7. RNA Sequencing and Bioinformatic Analysis

The watermelon varieties ZJU048 (waterlogging-sensitive) and ZJU068 (waterlogging-tolerant) were subjected to two treatments: a control treatment (normal water management) and a waterlogging treatment (continuous waterlogging for 14 days). Each treatment group consisted of three independent biological replicates. Following the treatment period, the basal 1–2 cm segment of the stem (encompassing the first internode region, including the shoot apical meristem and the elongation zone) was excised from each plant for RNA extraction. Total RNA was extracted from these samples and used for transcriptome sequencing and analysis. Sequencing libraries were subsequently constructed according to the following procedure. RNA purification was performed using Dynabeads Oligo (dT) (Thermo Fisher Scientific, Waltham, MA, USA). The purified RNA was fragmented using the NEBNext Magnesium RNA Fragmentation Module, and the resulting fragments were reverse-transcribed into first-strand cDNA. Double-stranded cDNA was synthesized, incorporating dUTP during the second-strand synthesis. The cDNA fragments were then size-selected and purified. Treatment with UDG enzyme was carried out to remove the dUTP-containing second strand. The resulting libraries were amplified by PCR and prepared for sequencing. High-throughput sequencing was performed on an Illumina NovaSeq 6000 platform (Illumina, Inc., San Diego, CA, USA) in 150 bp paired-end (PE150) mode. Raw sequencing reads were subjected to quality control (QC) using FastQC (V0.11.9) [23], and low-quality bases/adapters were trimmed using Trim-galore to generate clean read to generate clean reads. The clean reads from each sample were aligned to the watermelon reference genome (97103, v2.0) using the STAR aligner [32]. The genome annotation file (GFF format) was converted to GTF format using the gffread utility from Cufflinks (V2.2.1) [33,34], and this GTF file was supplied to STAR [32] for guided alignment, with output files generated in BAM format. The resulting BAM files were inspected using SAMtools (V1.10) [26]. Transcript abundance was estimated using RSEM (V1.3.3) [35], which generated read counts for each gene. These counts were compiled into an expression matrix. Differential expression analysis between sample groups was conducted using the DESeq2 (V1.42.0) [36] package in R. The results of the differential expression analysis were visualized using functions from the plotMA package. All plots were subsequently customized for clarity using the ggplot2 library, and gene labels were added with the assistance of the ggrepel package.

### 4.8. Cloning of the Candidate Genes

The full-length coding sequences (CDS) of the seven candidate genes within the fine-mapped interval (Cla97C07G134350–Cla97C07G134410, with a focus on Cla97C07G134400/ClaSCPL50) were amplified from cDNA of both ZJU048 and ZJU068 using gene-specific primers (Table S4). The amplified products were cloned into the pEASY-Blunt cloning vector (TransGen Biotech, Beijing, China) following the methodology established by Hu et al. [37], and verified by Sanger sequencing. All primers utilized in this process were designed with the NCBI Primer Designing Tool, as detailed in Table S4. Multiple sequence alignments of CDS and corresponding protein sequences were performed using Clustal X (V2.1) [38], and the resulting alignments were visualized and annotated with GeneDoc 2.7 software (https://genedoc.software.informer.com/ (accessed on 10 April 2026)).

### 4.9. Statistical Analysis

Data are presented as mean ± standard deviation (SD). A two-tailed Student’s *t*-test was used to assess significance for comparisons between two groups, while one-way analysis of variance (ANOVA) was applied for multiple group comparisons. Different letters denote a significant difference at *p* < 0.05. Differential expression analysis between two groups was conducted using the EdgeR package (V 4.11.0).

## 5. Conclusions

In this study, we identified *ClSCPL50* as a key candidate gene of the waterlogging escape response in watermelon. Natural variation in *ClSCPL50* between the two accessions, ZJU048 and ZJU068, provides a valuable target for molecular breeding aimed at enhancing waterlogging escape response—not only in watermelon but also potentially in other horticultural and cereal crops.

## Data Availability

The RNA-seq data in this study have been deposited in the CNCB BioProject under accession number CRA043333. The original contributions presented in this study are included in the article/Supplementary Materials. Further inquiries can be directed to the corresponding authors.

## Supplementary Materials

The following supporting information can be downloaded at: https://www.mdpi.com/article/10.3390/plants15111686/s1, Figure S1: Predicted domain architecture of the ClSCL50 protein; Figure S2: Alignment of promoter sequences of ClSCPL50 in ZJU048 and ZJU068; Table S1: Summary of sequencing data quality; Table S2: primers used for KASP genotyping; Table S3: primers used for qRT-PCR; Table S4: primer used for cloning of the candidate genes.

## References

[1] Jiménez J.d.l.C., Mustroph A., Pedersen O., Weits D.A., Schmidt-Schippers R. (2024). Flooding Stress and Responses to Hypoxia in Plants. Funct. Plant Biol..

[2] Li G., Wei N., Hou H. (2025). Uncovering the Secrets of How Plants Adapt to Water Stress. Plant Cell Environ..

[3] Xu K., Xu X., Fukao T., Canlas P., Maghirang-Rodriguez R., Heuer S., Ismail A.M., Bailey-Serres J., Ronald P.C., Mackill D.J. (2006). Sub1A Is an Ethylene-Response-Factor-like Gene That Confers Submergence Tolerance to Rice. Nature.

[4] Schmitz A.J., Folsom J.J., Jikamaru Y., Ronald P., Walia H. (2013). SUB 1 A -mediated Submergence Tolerance Response in Rice Involves Differential Regulation of the Brassinosteroid Pathway. New Phytol..

[5] Pierik R., Van Aken J.M., Voesenek L.A.C.J. (2009). Is Elongation-Induced Leaf Emergence Beneficial for Submerged Rumex Species?. Ann. Bot..

[6] Hattori Y., Nagai K., Furukawa S., Song X.-J., Kawano R., Sakakibara H., Wu J., Matsumoto T., Yoshimura A., Kitano H. (2009). The Ethylene Response Factors SNORKEL1 and SNORKEL2 Allow Rice to Adapt to Deep Water. Nature.

[7] Kuroha T., Nagai K., Gamuyao R., Wang D.R., Furuta T., Nakamori M., Kitaoka T., Adachi K., Minami A., Mori Y. (2018). Ethylene-Gibberellin Signaling Underlies Adaptation of Rice to Periodic Flooding. Science.

[8] Müller J.T., Van Veen H., Bartylla M.M., Akman M., Pedersen O., Sun P., Schuurink R.C., Takeuchi J., Todoroki Y., Weig A.R. (2021). Keeping the Shoot above Water—Submergence Triggers Antithetical Growth Responses in Stems and Petioles of Watercress (*Nasturtium officinale*). New Phytol..

[9] Nagai K., Mori Y., Ishikawa S., Furuta T., Gamuyao R., Niimi Y., Hobo T., Fukuda M., Kojima M., Takebayashi Y. (2020). Antagonistic Regulation of the Gibberellic Acid Response during Stem Growth in Rice. Nature.

[10] Chen W., Wang T., Li X., Feng J., Liu Q., Xu Z., You Q., Yang L., Liu L., Chen S. (2025). Arabidopsis RGLG1 /2 Regulate Flowering Time under Different Soil Moisture Conditions by Affecting the Protein Stability of TOE1/2. New Phytol..

[11] Cho N.H., Woo O.-G., Kim E.Y., Park K., Seo D.H., Yu S.G., Choi Y.A., Lee J.H., Lee J.-H., Kim W.T. (2022). E3 Ligase AtAIRP5/GARU Regulates Drought Stress Response by Stimulating SERINE CARBOXYPEPTIDASE-LIKE1 Turnover. Plant Physiol..

[12] Cho H., Choi I., Bouain N., Nawaz A., Zheng L., Shahzad Z., Brandizzi F., Rhee S.Y., Rouached H. (2026). Phosphorus Availability Controls Flowering Time through Subcellular Reprogramming of bGLU25 and GRP7 in Arabidopsis. Dev. Cell.

[13] Yetisir H., Çaliskan M.E., Soylu S., Sakar M. (2006). Some Physiological and Growth Responses of Watermelon [*Citrullus lanatus* (Thunb.) Matsum. and Nakai] Grafted onto *Lagenaria siceraria* to Flooding. Environ. Exp. Bot..

[14] Bailey-Serres J., Fukao T., Ronald P., Ismail A., Heuer S., Mackill D. (2010). Submergence Tolerant Rice: SUB1’s Journey from Landrace to Modern Cultivar. Rice.

[15] Voesenek L.A.C.J., Bailey-Serres J. (2015). Flood Adaptive Traits and Processes: An Overview. New Phytol..

[16] Fukao T., Yeung E., Bailey-Serres J. (2011). The Submergence Tolerance Regulator SUB1A Mediates Crosstalk between Submergence and Drought Tolerance in Rice. Plant Cell.

[17] Xu X., Ji J., Xu Q., Qi X., Weng Y., Chen X. (2018). The Major-effect Quantitative Trait Locus Cs ARN 6.1 Encodes an AAA ATP Ase Domain-containing Protein That Is Associated with Waterlogging Stress Tolerance by Promoting Adventitious Root Formation. Plant J..

[18] Du H., Zhu J., Su H., Huang M., Wang H., Ding S., Zhang B., Luo A., Wei S., Tian X. (2017). Bulked Segregant RNA-Seq Reveals Differential Expression and SNPs of Candidate Genes Associated with Waterlogging Tolerance in Maize. Front. Plant Sci..

[19] Mugford S.T., Milkowski C. (2012). Serine Carboxypeptidase-Like Acyltransferases from Plants. Methods in Enzymology.

[20] Tattersall D.B., Bak S., Jones P.R., Olsen C.E., Nielsen J.K., Hansen M.L., Høj P.B., Møller B.L. (2001). Resistance to an Herbivore Through Engineered Cyanogenic Glucoside Synthesis. Science.

[21] Sulpice R., Pyl E.-T., Ishihara H., Trenkamp S., Steinfath M., Witucka-Wall H., Gibon Y., Usadel B., Poree F., Piques M.C. (2009). Starch as a Major Integrator in the Regulation of Plant Growth. Proc. Natl. Acad. Sci. USA.

[22] Lyu X., Shi L., Zhao M., Li Z., Liao N., Meng Y., Ma Y., Zhou Y., Xue Q., Hu Z. (2022). A Natural Mutation of the NST1 Gene Arrests Secondary Cell Wall Biosynthesis in the Seed Coat of a Hull-Less Pumpkin Accession. Hortic. Res..

[23] Chen S., Zhou Y., Chen Y., Gu J. (2018). Fastp: An Ultra-Fast All-in-One FASTQ Preprocessor. Bioinformatics.

[24] Liao N., Hu Z., Li Y., Hao J., Chen S., Xue Q., Ma Y., Zhang K., Mahmoud A., Ali A. (2020). Ethylene-responsive Factor 4 Is Associated with the Desirable Rind Hardness Trait Conferring Cracking Resistance in Fresh Fruits of Watermelon. Plant Biotechnol. J..

[25] Li H., Durbin R. (2009). Fast and Accurate Short Read Alignment with Burrows–Wheeler Transform. Bioinformatics.

[26] Li H., Handsaker B., Wysoker A., Fennell T., Ruan J., Homer N., Marth G., Abecasis G., Durbin R. (2009). 1000 Genome Project Data Processing Subgroup. The Sequence Alignment/Map Format and SAMtools. Bioinformatics.

[27] McKenna A., Hanna M., Banks E., Sivachenko A., Cibulskis K., Kernytsky A., Garimella K., Altshuler D., Gabriel S., Daly M. (2010). The Genome Analysis Toolkit: A MapReduce Framework for Analyzing next-Generation DNA Sequencing Data. Genome Res..

[28] Wang K., Li M., Hakonarson H. (2010). ANNOVAR: Functional Annotation of Genetic Variants from High-Throughput Sequencing Data. Nucleic Acids Res..

[29] Takagi H., Abe A., Yoshida K., Kosugi S., Natsume S., Mitsuoka C., Uemura A., Utsushi H., Tamiru M., Takuno S. (2013). QTL -seq: Rapid Mapping of Quantitative Trait Loci in Rice by Whole Genome Resequencing of DNA from Two Bulked Populations. Plant J..

[30] Mansfeld B.N., Grumet R. (2018). QTLseqr: An R Package for Bulk Segregant Analysis with Next-Generation Sequencing. Plant Genome.

[31] Livak K.J., Schmittgen T.D. (2001). Analysis of Relative Gene Expression Data Using Real-Time Quantitative PCR and the 2^−ΔΔCT^ Method. Methods.

[32] Dobin A., Davis C.A., Schlesinger F., Drenkow J., Zaleski C., Jha S., Batut P., Chaisson M., Gingeras T.R. (2013). STAR: Ultrafast Universal RNA-Seq Aligner. Bioinformatics.

[33] Ghosh S., Chan C.-K.K., Edwards D. (2016). Analysis of RNA-Seq Data Using TopHat and Cufflinks. Plant Bioinformatics.

[34] Pertea G., Pertea M. (2020). GFF Utilities: GffRead and GffCompare. F1000Research.

[35] Li B., Dewey C.N. (2011). RSEM: Accurate Transcript Quantification from RNA-Seq Data with or without a Reference Genome. BMC Bioinform..

[36] Varet H., Brillet-Guéguen L., Coppée J.-Y., Dillies M.-A. (2016). SARTools: A DESeq2- and EdgeR-Based R Pipeline for Comprehensive Differential Analysis of RNA-Seq Data. PLoS ONE.

[37] Hu Z., Deng G., Mou H., Xu Y., Chen L., Yang J., Zhang M. (2018). A Re-Sequencing-Based Ultra-Dense Genetic Map Reveals a Gummy Stem Blight Resistance-Associated Gene in Cucumis Melo. DNA Res..

[38] Larkin M.A., Blackshields G., Brown N.P., Chenna R., McGettigan P.A., McWilliam H., Valentin F., Wallace I.M., Wilm A., Lopez R. (2007). Clustal W and Clustal X Version 2.0. Bioinformatics.

