# Supplementary figures and images for: ClSCPL50 Is Associated with Waterlogging-Induced Shoot Elongation in Watermelon as Revealed by BSA-Seq and Transcriptome Profiling
Source: Plants (Basel). 2026 May 29;15(11):1686. doi: 10.3390/plants15111686 (PMC13259023; doi:10.3390/plants15111686)

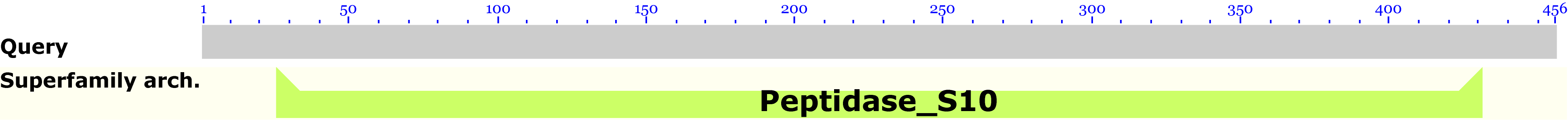

Supplement: Supplementary file 1 [file plants-15-01686-s001.zip › Figure S1-ClSCPL50_domain.tif]

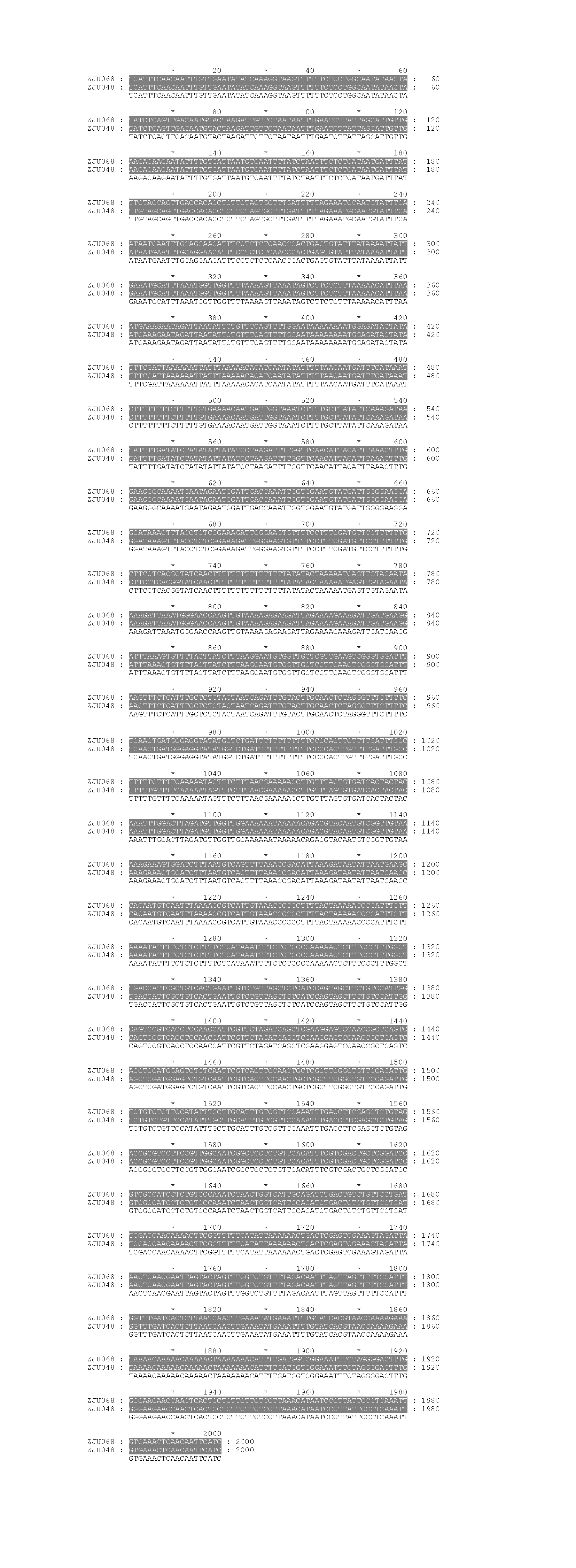

Supplement: Supplementary file 1 [file plants-15-01686-s001.zip › Figure S2-Alignment of promoter sequences of ClSCPL50 in ZJU048 and ZJU068..tif]
